# METTL16, Methyltransferase-Like Protein 16: Current Insights into Structure and Function

**DOI:** 10.3390/ijms22042176

**Published:** 2021-02-22

**Authors:** Agnieszka Ruszkowska

**Affiliations:** Department of Structural Chemistry and Biology of Nucleic Acids, Institute of Bioorganic Chemistry, Polish Academy of Sciences, 61-704 Poznan, Poland; aruszkowska@ibch.poznan.pl

**Keywords:** METTL16, m^6^A methyltransferases, SAM homeostasis, U6 snRNA

## Abstract

Methyltransferase-like protein 16 (METTL16) is a human RNA methyltransferase that installs m^6^A marks on U6 small nuclear RNA (U6 snRNA) and *S*-adenosylmethionine (SAM) synthetase pre-mRNA. METTL16 also controls a significant portion of m^6^A epitranscriptome by regulating SAM homeostasis. Multiple molecular structures of the N-terminal methyltransferase domain of METTL16, including apo forms and complexes with *S*-adenosylhomocysteine (SAH) or RNA, provided the structural basis of METTL16 interaction with the coenzyme and substrates, as well as indicated autoinhibitory mechanism of the enzyme activity regulation. Very recent structural and functional studies of vertebrate-conserved regions (VCRs) indicated their crucial role in the interaction with U6 snRNA. METTL16 remains an object of intense studies, as it has been associated with numerous RNA classes, including mRNA, non-coding RNA, long non-coding RNA (lncRNA), and rRNA. Moreover, the interaction between METTL16 and oncogenic lncRNA MALAT1 indicates the existence of METTL16 features specifically recognizing RNA triple helices. Overall, the number of known human m^6^A methyltransferases has grown from one to five during the last five years. METTL16, CAPAM, and two rRNA methyltransferases, METTL5/TRMT112 and ZCCHC4, have joined the well-known METTL3/METTL14. This work summarizes current knowledge about METTL16 in the landscape of human m^6^A RNA methyltransferases.

## 1. Introduction

To date, 143 types of RNA modifications are known to exist in the three domains of life [1]. One of the most abundant and well-studied modifications, *N*^6^-methyladenosine (m^6^A), has been identified in all RNA classes. m^6^A affects multiple aspects of RNA processing, including biogenesis, stability regulation, half-life control, pre-mRNA splicing, export, and translation [2,3,4,5,6,7,8]. Dynamic m^6^A pattern of transcriptome influences a number of biological processes and requires orchestrated cooperation of adenosine methyltransferases (“writers”), proteins recognizing m^6^A marks (“readers”), and m^6^A-demethylating enzymes (“erasers”) [9].

The field of structural and functional studies of human m^6^A writers is rapidly growing. In eukaryotes, the METTL3/METTL14 complex installs most m^6^A marks on mRNA [10]. A series of studies have revealed that the METTL3/METTL14 complex is assisted by WTAP, RBM15, VIRMA, ZC3H13, and HAKAI proteins, which recruit the complex to its target sites [11,12,13,14,15]. The last few years have brought to our attention several new human m^6^A methyltransferases. METTL16 methylates U6 small nuclear RNA (U6 snRNA), MAT2A mRNA encoding *S*-adenosylmethionine (SAM) synthetase, and possibly other RNAs. Moreover, METTL16 has been linked to numerous m^6^A modifications in the epitranscriptome due to its role in regulation of SAM homeostasis [16,17,18].

A recently discovered cap-specific adenosine methyltransferase (CAPAM) PCIF1 distinctly recognizes 2′-O-methyladenosine (Am) if it is the first transcribed nucleotide of eukaryotic capped mRNAs and installs the m^6^A mark to form m^7^Gpppm^6^Am motif [19,20]. Human m^6^A methyltransferases METTL5/TRMT112 complex and ZCCHC4 modify A1832 of 18S and A4220 of 28S rRNA, respectively [21,22,23]. 

The bacterial rRNA is methylated by RlmF, a homolog of human METTL16, and RlmJ. These two methyltransferases install m^6^A marks in *Escherichia coli* 23S ribosomal subunit at the positions A1618 and A2030, respectively [24,25]. TrmM confers the m^6^A methylation in tRNA^Val^ in *E. coli* [26]. Despite the presence of m^6^A in bacterial mRNA, enzymes associated with these marks are unknown [1].

The homologs of METTL16 have been determined in organisms ranging from bacteria to mammals. METT-10, found in *Caenorhabditis elegans*, is a nuclear protein involved in inhibiting germ-cell proliferative fate and promoting mitotic cell cycle division [27]. The *Arabidopsis thaliana*, FIONA1 regulates period length in the circadian clock [28]. 

Human METTL16, the object of this review, has been under the scope for the last five years and the understanding of this protein has grown significantly during this time. The interest in METTL16 is due to multiple reasons. It has sequential and structural requirements for substrates. Simultaneously, METTL16 affects a broad m^6^A landscape in the transcriptome, representing sites not complying with these requirements. METTL16 seems to function as m^6^A “reader” to regulate splicing of SAM synthetase transcript. Thus, it guards SAM homeostasis in the cell. Moreover, METTL16 interacts with multiple RNAs; for instance, it specifically recognizes the RNA triple helix of oncogenic long non-coding RNA (lncRNA) MALAT1; however, the role of these interactions remains elusive.

## 2. Structure of the Human METTL16

**Figure 1:**
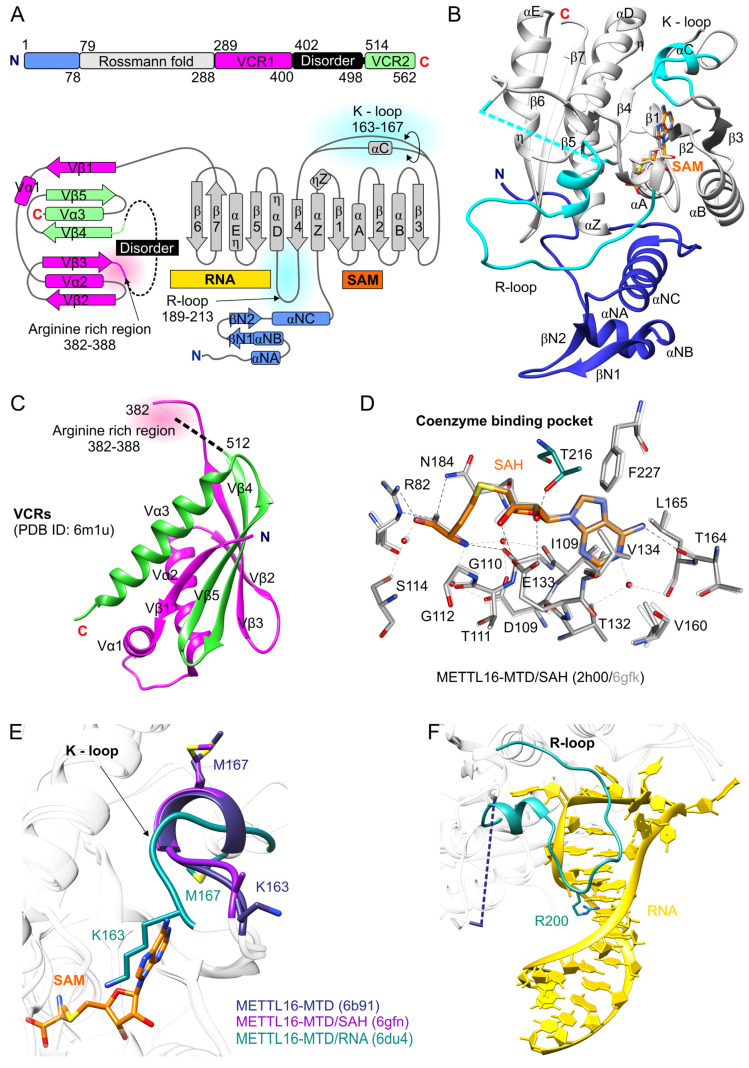
The structure of METTL16. (**A**) Scheme of the overall domains’ organization and topology diagram of human METTL16 [29,36] (**B**) The 3D structure of METTL16 is a composite of three superposed models: apo-METTL16-MTD (PDB ID: 6b91), METTL16-MTD/SAH (6gfn), METTL16-MTD/RNA (6du4)—RNA is not shown; METTL16 is shown as ribbons and structural elements are color-coded as in panel A. Dashed cyan line represents disordered residues 189–213 (R-loop) in apo form of METTL16-MTD. (**C**) Crystal structure of vertebrates conserved regions (VCRs) located in the C-terminal domain of METTL16; the arginine-rich region is indicated. The dashed black line represents predicted disordered residues 402–498. (**D**) The network of interactions secures the orientation of SAH in the METTL16 coenzyme binding pocket. The model presents interactions in the 2h00 structure (residues-gray, SAH-orange) and superposed 6gfk (transparent gray). Residue T216 (turquoise) is modeled from METTL16-MTD/hp1x (6du4) structure. Black dashed lines show direct interactions between METTL16 and the coenzyme, contacts mediated by water molecules (red/gray dots) are shown by gray dashed lines. (**E**) Conformational rearrangement of the K-loop between apo-METTL16-MTD (6b91, dark purple), METTL16-MTD/SAH (6gfn, dark pink) and METTL16-MTD/hp1x (6du4, turquoise) complexes. Upon RNA binding K163 is placed inside the SAM binding pocket. The K-loop is highlighted and shown by ribbon representation; crucial residues of this motif, K163 and M167, are presented as sticks. (**F**) The R-loop in the METTL16-MTD/hp1x complex (6du4, turquoise) is shown in ribbon representation. R200 is shown as sticks. Dashed dark purple line represents the disordered R-loop existing in the apo structure of METTL16. All molecular figures were created using UCSF Chimera [37].

Human METTL16 [UniProt ID: Q86W50] is 562 amino acid residues long. It contains the N-terminal methyltransferase domain (MTD) conserved from *Escherichia coli* through humans and the C-terminal domain. The latter is composed of two vertebrate-conserved regions (VCRs), spanning residues 289–400 and 514–562, and also contains disordered evolutionary variable residues 402–498 (Figure 1A–C) [16,29]. There is still inconsistency in the reports determining the oligomeric state of METTL16. The METTL16 ortholog in *C. elegans*, METT-10 is a homodimer [30]. Moreover, size-exclusion chromatography (SEC) coupled with small-angle X-ray scattering (SAXS) has indicated that full-length human METTL16 exists as a dimer, while MTD (region 1–291) is a monomer [29]. However, subsequent experiments, including SEC and SEC followed by multi-angle light scattering (MALS), showed that METTL16 is a monomer in solution [31,32]. The determination of molecular weight (MW) using SAXS data should be accurate enough to evaluate an oligomeric state; however, the SAXS MW calculations are error-prone (≥10%) [33,34]. Since MALS measurements are independent of biomolecule shape and define the absolute molecular mass [35], this technique is more reliable than SAXS in determining the oligomeric state. Thus, METTL16 is most likely monomeric. 

### 2.1. N-Terminal Domain of METTL16 Has m^6^A Methyltransferase Activity

METTL16 belongs to the class I SAM-methyltransferases (SAM-MTases), which contain the Rossmann fold. The conserved core of METTL16 includes residues 79–288. The core is composed of a seven-stranded β-sheet (β strands in order 3214576), placed between clusters of α and 3_10_ (η) helices. The β-strands and helices, alternating in the secondary structure of the Rossmann fold, create SAM and RNA binding sites within the N-terminal and C-terminal segments of the β-sheet, respectively (Figure 1A,B) [29,38]. SAM interactions with METTL16 were determined based on the crystal structures of METTL16-MTD/SAH complexes (PDB ID: 2h00, 6b92, 6gfn, 6gfk) [29,32]. The network of hydrogen bonds, both direct and indirect (involving water molecules), secure the proper position of the coenzyme in the pocket of METTL16 (Figure 1D). The adenine moiety of SAM/SAH is situated in a hydrophobic environment created by I109, V134, V160, L165, and F227 residues and makes a direct H-bond with T164. A conserved in class I SAM-MTases E or D residue (E133 in METTL16) [38,39] interacts with 2′- and 3′-hydroxyl groups of the ribose. Moreover, based on the crystal structure of METTL16-MTD/RNA (6du4), T216, disordered in METTL16-MTD/SAH bound structures, likely binds with O2′ (Figure 1D) [31]. 

The GXG motif, conserved in class I SAM-MTases [38,39], is represented in METTL16 by residues G110-T111-G112, which shape the cavity between adenosine and homocysteine/methionine moieties of SAH/SAM; however, only G110 makes direct contact with the coenzyme (Figure 1D). Mutation of G110 to C, identified in large intestinal cancer patients, abolishes in vitro methylation activity. A similar effect has been observed for E133A substitution (Table 1) [31]. The homocysteine/methionine moiety is recognized by R82 and N184, the first residue of the NPPF catalytic motif in METTL16 (Figure 1D). The side chain of N184 is flexible, as its orientation varies in METTL16-MTD/SAH complexes (2h00, 6gfk, 6gfn) and structures without SAH/SAM (6b91, 6du4). Upon the coenzyme binding, the side chain of N184 reorients to prime the acceptor adenosine for methyl transfer [29]. Mutations of R82 and N184 to alanine cause loss of m^6^A methyltransferase activity, indicating their crucial role in methylation (Table 1) [31,32]. 

The N-terminal domain (1–291 aa, MTD) of METTL16 alone shows methyltransferase activity towards the identified RNA substrates of METTL16, hairpins (hp1-hp6) of MAT2A mRNA and (less efficiently compared to full-length METTL16) U6 snRNA [16,31,36]. Even though the methyltransferase domain of METTL16 reveals the canonical structure of class I SAM-MTases, unique regions important for interaction with specific RNA substrates may be indicated [29,31]. First, METTL16 contains a unique 1–78 region, including three α helices and two short β strands, which precede the Rossmann fold (Figure 1A,B) [29,32]. This region seems essential for interaction with RNA substrates since the truncated METTL16-MTDΔN protein (residues 40–291) does not bind and methylate MAT2A hairpin hp1. Moreover, positively charged residues K5, R10, R12, K14, and K16 within N-terminus appear to play a role in RNA substrate accommodation as a combined mutation of these residues to alanines completely abolishes methyltransferase activity and highly reduces RNA binding (Table 1) [32]. However, none of the above residues makes direct contact with RNA in METTL16-MTD/RNA complex (6du4) [31]. Most of the residues (except K5) are away from the bound RNA and contribute to the global architecture of METTL16. Their involvement in substrate recognition may be more complex and perhaps include stepwise RNA binding.

Superposition of METTL16-MTD apo form (PDB ID: 6b91), METTL16-MTD/SAH complex (6gfn), and METTL16-MTD/RNA complex (6du4) reveals structural differences in the 163–167 region, the so-called K-loop between the β3 and β4 strands. The K-loop actually forms a short α helix (αC) in structures non-bound to RNA substrate (6b91, 6gfn), while in the METTL16-MTD/RNA complex (6du4), it functions as a loop (Figure 1A,B,E). Importantly, in contrast to apo and SAH-bound structures where K163 is solvent-exposed, in the METTL16-MTD/RNA complex, K163 is placed inside the SAH/SAM binding pocket, disrupting the coenzyme binding (Figure 1E). Similarly, a distinctly different orientation was observed for M167 (Figure 1E) [29,31]. Substitutions K163A and M167A significantly increase methylation activity with little effect on RNA affinity (Table 1). Thus, the K-loop seems to play an autoregulatory role in METTL16 activity. It is possible that the interaction of METTL16 with RNA substrates and autoinhibitory rearrangement of the K-loop are tied together. However, it is not known how RNA binding could affect structural changes of K-loop [31].

The long loop 189–213 located between the αD helix and the β4 of the METTL16 Rossmann fold (Figure 1A,B,F) interacts with the RNA substrate and affects m^6^A methylation [31]. For clarity, the region 189–213 hereafter will be referred to as the R-loop. In METT-10, the corresponding loop is longer and reveals low sequence conservation in comparison to its vertebrate orthologs [32]. The R-loop is disordered in METTL16-MTD structures non-bound with RNA [29,32]. Upon RNA binding, the R-loop adopts a defined structure, observed in the METTL16-MTD/substrate complexes (PDB ID: 6du4, 6du5 Figure 1F) [31]. Importantly, deletion of residues 190–218 causes loss of in vitro methylation activity (Table 1). Mutations of three positively charged residues, R200, R203, and R204 to glutamates abolish METTL16 methylation [32]. Interestingly, the single mutation R200Q significantly increases m^6^A modification efficiency (Table 1). R200 is located at the apex of the R-loop (Figure 1F). This residue interacts with the RNA substrate and seems to stabilize its conformation (6du4, 6du5, see section: Structural basis of METTL16 interaction with RNA methylation substrates) [31]. It is possible that the different effects of R200Q and R200E mutations on METTL16 methylation activity are due to an altered RNA affinity. Hypothetically, a weaker interaction between METTL16 and RNA in the R200Q mutant could facilitate disassociation of protein/RNA complex and accelerate the enzyme turnover. On the other hand, the R200E mutation would cause repulsion of the RNA substrate, and this way abolish methylation. Consistently, RRR-200-203-204-EEE mutation highly reduces RNA binding [32]. However, mutation R200Q or even deletion of the 190–218 loop do not disturb RNA binding (Table 1) [31,32], indicating that R200 has another role a step after initial binding of the RNA. In summary, R200 appears to be the critical residue of the R-loop, supporting the conformation of RNA substrate and tuning methylation activity of METTL16 [31]. 

### 2.2. The C-Terminal Domain of METTL16 Consists of a Long Disordered Region and VCRs

So far, determination of METTL16 C-terminal domain complete structure was not possible due to the long disordered region spanning residues 402–498 [29]. However, a recently solved crystal structure of the VCR_ΔL construct, representing residues 310–410 and 509–562 (PDB ID: 6m1u), shed light on the VCRs architecture and function (Figure 1A,C) [36]. An earlier study had indicated that the VCRs are involved in regulating MAT2A mRNA splicing [16].

The VCRs structure resembles kinase-associated 1 (KA1) domains found in various proteins [40], including the U6 snRNA-specific terminal uridylyltransferase (TUT1) [41]. The VCRs of METTL16 likely target the double-stranded RNA but their sequence specificity, if any, is not known [36]. Interaction of the VCRs with U6 snRNA seems to stabilize the proper conformation of the RNA substrate and enhance methylation catalyzed by the MTD. Functional studies of the VCRs revealed that the arginine-rich region, spanning residues 382–388 (Figure 1A,C), is crucial for RNA binding and methylation. Deletion of that region reduced methylation to the level detected for MTD alone (Table 1) [36]. However, the VCRs in trans do not increase MTD activity. This indicates some dependency between MTD and VCRs of METTL16. Interestingly, VCRs could be functionally replaced with KA1; the chimeric protein MTD-METTL16 + KA1 is more active towards U6 substrate than MTD alone. Thus, the VCR and KA1 share common functions to bind RNA and promote various steps of U6 snRNA biogenesis [36].

## 3. RNA Methylation Substrates of Human METTL16

Genome-wide studies have identified many RNA sites of METTL16 binding [16,17,42]. However, given that the methylation substrate of METTL16 has to meet very specific sequential and structural requirements [16,31,32], the number of targets modified efficiently by METTL16 is probably significantly smaller. So far, two RNA substrates of human METTL16 have been confirmed: MAT2A transcript encoding SAM synthetase and U6 snRNA [16,17,32,43]. Both targets contain a conserved UACAGAGAA sequence (methylated A is underlined), recognized by METTL16. METTL16 has been proposed as a conserved eukaryotic U6 snRNA methyltransferase [16]. A homolog of METTL16 (Duf890) has been identified in *Schizosaccharomyces pombe*, whose U6 snRNA is m^6^A methylated [44]. Moreover, deletion of Duf890 results in slow-growing yeast with loss of U6 m^6^A methylation [16]. In contrast, *Saccharomyces cerevisiae* apparently neither has a METTL16 homolog nor contains m^6^A on U6 snRNA [16].

The U6 snRNA m^6^A43 methylation by human METTL16, has been confirmed in vitro and in cellulo [16,17]. METTL16 likely interacts with nascent U6-RNA or U6 mono-snRNP at the early steps of U6 maturation [17]. Interestingly, three proteins involved in the biogenesis of mature U6 snRNA are partners of METTL16 [17], namely (i) guanosine triphosphate capping enzyme MEPCE [45], (ii) the chaperone-like La protein [46], and (iii) the La-associated protein LARP7 [47]. The latter two favorably bind oligo(U) stretches at the 3′ ends of RNA polymerase III transcripts [17,48,49,50]. The METTL16 interactions with these proteins are RNA-dependent. Warda et al. suggest that a nucleoplasmic, 5′-capped, oligouridylated pre-U6 snRNA form, stabilized by La, LARP7, and MEPCE, interacts with METTL16 [17]. 

The modified A43 of U6 snRNA [51,52] lies in a highly conserved sequence ACm^6^AGAGA involved in the interaction with 5′ splice site of pre-mRNA [53,54,55,56,57]. In yeast, mutations within this conserved motif are lethal, suggesting the importance of A43 for the regulation of pre-mRNA splicing [58]. Detailed functions of m^6^A43 modification remain vague. However, based on the structures of human U4/U6.U5 tri-snRNP and various (pre-)catalytic spliceosomes, the m^6^A43 mark seems irreversible. Furthermore, it appears to affect base pairing or local secondary structure of the U6 snRNA rather than being a reader protein target [17]. Given that the entire assembly of the spliceosome/pre-mRNA complex is governed by subtle interactions, m^6^A43 is suggested to influence snRNA–pre-mRNA contacts and regulate either spliceosome assembly or recognition of 5′ splice site [17].

METTL16 installs methylation marks within the conserved UACm^6^AGAGAA sequence in mRNA MAT2A 3′UTR hairpins, evolutionarily conserved among vertebrates [16,43,59]. The specific methylations of the six hairpins affect the splicing and stability of MAT2A pre-mRNA, regulating SAM homeostasis. The RNA hairpin hp1 controls intron retention. When the SAM supply is limited, METTL16 has a slower turnover and halts on hp1 localized in the last intron’s proximity [16,43]. This prolonged occupancy of METTL16 on hp1 promotes splicing of the transcript. The METTL16-dependent splicing induction is likely a co-transcriptional event. In the availability of SAM, hp1 is rapidly methylated, and METTL16 disassociates from the complex. Consequently, the intron is retained in the pre-mRNA form, and the nuclear MAT2A becomes a subject for RNA decay involving PABPn1-PAPα/γ [16,60,61]. Notably, modulating the METTL16 methyltransferase activity influences splicing of MAT2A transcript. Hyperactive METTL16 mutants (K163A or R200Q) cause intron retention even at low SAM levels. In contrast, catalytically inactive METTL16 mutant (N184A) induces mRNA splicing regardless of SAM availability (Table 1). Thus, METTL16 residues located in the K-loop or interacting with the RNA substrate are essential for tuning METTL16 to maintain a proper physiological level of SAM [31].

The MTD of METTL16 alone can install m^6^A on hp1 MAT2A. Nevertheless, the MTD is not sufficient to drive splicing. Induction of pre-mRNA MAT2A splicing requires VCRs of METTL16, and these domains seem to have evolved together with MAT2A hairpins [16]. The hairpins hp2-hp6 of MAT2A, also targeted by METTL16, do not affect splicing. In an abundance of SAM, these structures are modified and promote destabilization of the MAT2A transcript and its degradation [16,43]. The YTHDC1 m^6^A reader appears to also contribute to MAT2A mRNA stability control. However, preliminary results indicate that this protein may be involved in the processing of a mature MAT2A transcript without the intron [43]. The molecular mechanism of YTHDC1-dependent regulation of MAT2A processing remains elusive.

## 4. Search for Other RNA Partners of METTL16

METTL16 contributes to the total m^6^A methylation pattern in the cell. It has been reported that ~20% of total m^6^A marks in 293A-TOA cells are not installed upon METTL16 knockdown. However, the METTL16-dependent m^6^A modifications generally are not related to UACAGAGAA motif [16]. One explanation of the altered m^6^A pattern is that the activity of METTL16 may expand to other than UACAGAGAA targets through unknown cellular co-factors. Another possibility is linked to the fact that METTL16 affects the SAM level by regulating the expression of MAT2A. When SAM availability is depleted, total methylation in the cells drops [16]. In fact, Mendel et al., in studies on mice, indicated that METTL16 influences early embryo development by regulating SAM synthetase expression [32]. The MAT2A mRNA is the single key target of the enzyme in pre-implantation embryos. Knockout of METTL16 causes downregulation of MAT2A mRNA, and downstream epigenetic reprograming events fail due to disrupted SAM homeostasis [32]. Similarly, in 293A-TOA or HEK293T cells, target sites of METTL16 seem to be indirect, since (i) MAT2A knockdown and overexpression have an antagonistic effect on methylation of METTL16-dependent sites—decreasing and increasing, respectively; (ii) putative substrates are not bound in cellulo and are not methylated by METTL16 in vitro; and (iii) multiple METTL16-dependent m^6^A marks were co-identified as METTL3- or PCIF1-dependent modifications [16,18]. However, MAT2A overexpression in cells does not completely complement METTL16 depletion. Thus, the reduced level of METTL16-dependent m^6^A marks is not entirely the result of limited SAM availability; a more direct effect of METTL16 may be involved [16].

METTL16 interacts with rRNA, non-coding RNAs (ncRNA), lncRNAs, and numerous mRNAs [16,17,42,43,62]. Notably, 93% of crosslinks in mRNAs were detected within introns, suggesting that METTL16 interacts with a subset of pre-mRNAs [17]. Interestingly, among the determined METTL16 partners are MALAT1 (metastasis-associated lung adenocarcinoma transcript 1), which is an oncogenic lncRNA [17,42,63], and XIST (X-inactive specific transcript) lncRNA involved in the inactivation of the X chromosome [17,64,65]. According to Warda et al., approximately 10%, 25%, and 83% of identified METTL16 crosslinking sites overlap with the m^6^A marks found in mRNAs, lncRNAs, and ncRNA, respectively [17]. However, the precise identification of METTL16-associated methylation positions is still problematic due to the following reasons. First, a comparison of datasets reporting m^6^A pattern in transcriptome reveals relatively low overlap, suggesting that only a portion of methylated sites have been mapped. Second, some methods for m^6^A detection rely on the determination of the RAC sequence, specific for METTL3 but suboptimal for METTL16. Third, pre-mRNAs are under-represented in total RNA compared to mature mRNA, which causes underestimation of m^6^A sites placed in introns [17].

Bioinformatic analysis of METTL16 functional relevance has shown that many METTL16-dependent m^6^A sites occur in genes related to the endoplasmatic reticulum-associated misfolded protein catabolism, regulation of protein transport and ubiquitination, apoptosis, cell cycle, DNA-templated transcription, and actin cytoskeleton organization [16,66]. Additionally, METTL16 is an element of the UV- induced DNA damage response, wherein METTL16 appears to be responsible for m^6^A modification of small RNAs (snRNAs and small nucleolar RNAs) near the DNA lesions [67]. 

A few new putative mRNA methylation substrates of METTL16 have been proposed: RBM3, STUB1, and ISYNA1 [17]. These three partners of METTL16 contain m^6^A marks within the METTL16 crosslinking sites and the marks are reduced by METTL16 knockdown [16,17]. However, given that METTL16 regulates SAM homeostasis, many METTL16-related methylations are affected by SAM availability rather than are direct targets of the enzyme. Moreover, METTL16 involvement in splicing of the MAT2A transcript, irrespective of catalytic activity [16], indicates that some METTL16-associated RNAs may not undergo METTL16 methylation, and their interaction with the protein could have another biological function.

Initially, METLL16 has been determined as a nuclear protein. This localization is consistent with functions so far reported for METTL16, such as methylation of MAT2A pre-mRNA and U6 snRNA, MAT2A splicing regulation, and interaction with lncRNAs (XIST, MALAT1), and introns [17,42]. However, recent studies by Nance et al. indicated that at least 50% of METTL16 localize in the cytoplasm of different cell lines (HEK293T, HELA, lung fibroblast CCD34LU, and cancer cell lines: NCI-H1299, and series lines of MCF10). The role of METTL16 in the cytosol remains unclear. However, the authors suggest that the altered subcellular localization of METTL16 may affect RNA-binding preferences [62].

### METTL16 and the 3′ Triple Helical Structure of MALAT1 lncRNA

METTL16 recognizes the RNA triple helix located at the 3′ end of cancer-promoting MALAT1 [17,42]. The U-rich internal loop at the 3′ end of MALAT1 associates with a downstream genomically encoded A-rich stretch to form a bipartite triple helix composed of canonical triples: nine U•A-U, one C•G-C, and a C-G doublet. The triple helical structure stabilizes MALAT1 and allows it to accumulate in cells [68]. Interaction between METTL16 and triple helix depends on both the structure and nucleotide composition of the latter [42]. However, the role of this interaction remains elusive. It is unknown whether the association of METTL16 and MALAT1 involves methylation. The RNA triple helix does not bind efficiently with the MTD domain alone [29]. However, because the full-length protein interacts with MALAT1 [29], the VCRs could facilitate MALAT1 binding and methylation, as shown for U6 snRNA [36]. A weak m^6^A mark was found at the A8290 position in the RNA duplex near the triple helical structure of MALAT1. Nevertheless, the sequential context of this modification (CAm^6^ACA) appears suboptimal for METTL16. Understanding the nature of METTL16/MALAT1 interaction is important for at least two reasons. First, insight into the structural basis of MALAT1/METTL16 interaction could reveal a protein motif specialized for recognizing RNA triple helices. Second, since (i) human METTL16-dependent m^6^A marks are associated with genes related to apoptosis regulation, (ii) METTL16 homolog from *C. elegans* affects cell proliferation, and (iii) MALAT1 promotes oncogenesis, the METTL16/MALAT1 interaction could be linked to carcinogenesis.

## 5. Structural Basis of METTL16 Interaction with RNA Methylation Substrates

**Figure 2:**
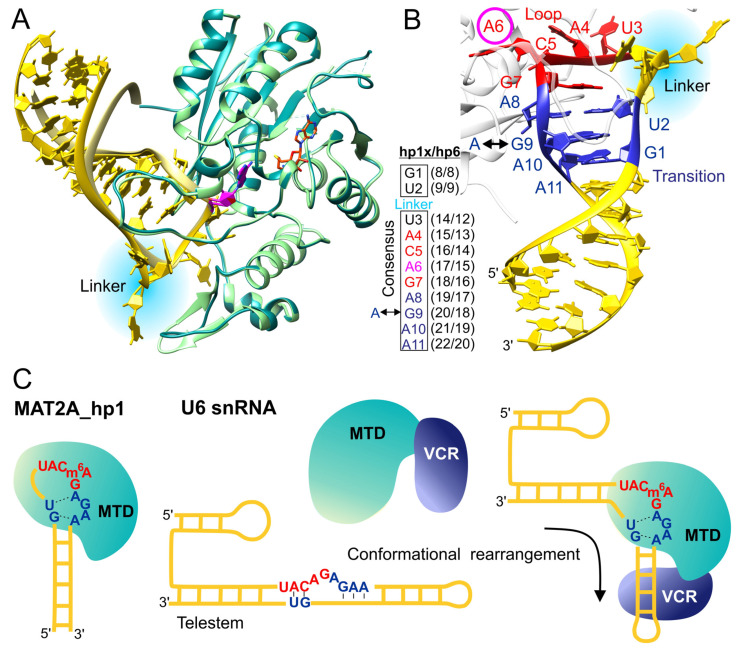
Interaction between METTL16 and RNA methylation substrates. (**A**) Superposition of two crystal structures of METTL 16-MTD/RNA complex, PDB ID: 6du4 (METTL16, turquoise; hp1x, gold), 6du5 (METTL16, light green; hp6, light yellow); the acceptor adenosine is shown in magenta; SAH is superimposed from the structure 6b92 and shown as sticks (orange). The linker differentiating the loop-transition region of hp1x and hp6 is highlighted by light blue. (**B**) The hp1x of METTL16-MTD/RNA complex (6du4) is shown in atoms-ribbon representation. The 3′ part of the loop and the transition region are marked by red and blue colors, respectively. The acceptor adenosine is manifested by the magenta circle. The linker between the transition region and the loop is highlighted by light blue. METTL16 is shown by a transparent white ribbon. The conserved sequence of the loop-transition structure is numbered 1–11, with 6 (magenta) representing the acceptor adenosine. Schematic representation of loop-transition region (inset) shows conserved residues (1–11, box) and corresponding nucleotides in hp1x and hp6 models. The arrow indicates the G/A substitution in hp5. (**C**) A schematic diagram of the interaction between METTL16 and its RNA substrates ([36], figure modified): MAT2A mRNA and U6 snRNA. The loop and transition regions are marked as in panel B. The VCRs of METTL16 associate with U6 and rearrange the substrate to obtain the conformation optimal for methylation.

Recently, Doxtader et al. have solved the crystal structure of the methyltransferase domain of METTL16 (1–310 aa) in a complex with RNA substrates, hp1 (hp1x, PDB ID: 6du4, Figure 2A) and hp6 (6du5, Figure 2A) [31]. These two structures have significantly deepened our understanding of the recognition manner between RNA and METTL16 as well as revealed structural dependencies of substrate and enzyme in tuning methyltransferase activity of METTL16. The substrate RNA, hp1x or hp6, binds within the positively charged groove of METTL16 at 1:1 ratio. An extensive network of interactions involving three polypeptide segments of METTL16 (residues 34–48, 189–213 [R-loop], 277–280) ensures the specific substrate recognition. The target adenine is positioned in the hydrophobic pocket near the catalytic motif NPPF (residues 184–187). Comparison of the 184-NPPFF-188 motif conformations in METTL16-MTD/RNA complex and in METTL16-MTD without RNA shows that upon substrate binding, F187 and F188 shift to interact with the acceptor adenosine. In other words, the formation of the METTL16/RNA complex involves an induced-fit mechanism, where some regions of METTL16 change conformation to ensure adequate contacts between the enzyme and the RNA substrate [31].

### 5.1. The Loop-Transition Structure of RNA Substrate Modulates METTL16 Methylation

Substrate specificity for METTL16 binding and activity has been characterized. Both the conserved consensus UACAGAGAA sequence and the structure of RNA play crucial roles in the efficient m^6^A writing by METTL16 [16,31,32,59]. The hp1x and hp6 substrates are composed of three regions: recognition loop, transition region, and stem (Figure 2A,B). Bases within the substrate loop are exposed outside, and the 3′ part of the loop (3-UACAG-7, red, numbering in Figure 2B) maximizes sequence-specific contacts with METTL16 by hydrogen bonding and stacking. Point mutations of these nucleotides significantly decrease in vitro methylation but only modestly affect affinity to METTL16. Likely, those base substitutions influence events after the complex formation [31]. 

The transition structure of hp1x and hp6 substrates lies between the loop and the stem. Its proper conformation is ensured by the 5′- and 3′-conserved motifs (1-GU-2, and 8-AGAA-11, respectively; blue, Figure 2B). The transition region is composed of: (i) the U2-A8 pair, exhibiting glycosidic bonds in *trans* conformation, (ii) G9-A10, hydrogen bonding via an ordered water molecule, and (iii) the stem-preceding A11-G1 pair, which makes contacts by their Hoogsteen and sugar edges, respectively. R200 located within the R-loop of METTL16, is involved in extended interactions with the transition region. R200 hydrogen-bonds to G1 and G9, stabilizing the transition structure [31].

The six hairpins (hp1-6) of 3′UTR MAT2A mRNA exhibit different predispositions for methylation by METTL16 in vitro [31]. Hp1 is methylated more efficiently than hp6. However, substitution of the stem regions between hp1 and hp6 does not significantly change the METTL16 binding and m^6^A writing activity. Thus, it is the loop-transition structure that modulates the in vitro methylation efficiency [31]. Alignment of the hairpin sequences reveals differences in the loop-transition region. First, the linker between the two conserved blocks (GU-linker-UACAGAGAA) alters in nucleotide composition and length, which may modulate METTL16 activity. This element connects the 5′ part of the transition region and the 3′ end of the loop (Figure 2A,B). A second variation is the G9A substitution (Figure 2B) within the transition region of hp5 (UACAGAAAA), which enhances methylation in vitro. Interestingly, G-to-A mutation within the transition region of hp1 and hp6, corresponding to the wild type sequence of hp5, increases in vitro methyltransferase activity of METTL16. A similar effect was observed for R200Q substitution; R200 is the residue in METTL16 that stabilizes the conformation of the transition structure. Both mutations, G9A in RNA and R200Q in METTL16, have a slight effect on RNA affinity [31]. Altogether, the transition region seems crucial in tuning the methylation efficiency. Modifications within this structure are likely to affect the METTL16 m^6^A writing activity at a stage after the initial binding of the RNA substrate. Possible explanations for the impact of the transition structure on methylation are (i) a faster disassociation of methylated substrate, (ii) an allosteric effect facilitating SAM binding or methyl transfer, (iii) an allosteric effect favoring SAH release, and (iv) a stable alternative conformation of transition structure caused by mutation/s [31].

The stem of the RNA substrate is not involved in direct contact with the N-terminal domain of METTL16. However, it is likely that the dsRNA region stabilizes the transition structure, as deletion of the stem disrupts the complex formation and in vitro methylation activity of METTL16 [31]. Recent studies of METTL16 VCRs (Figure 1C) indicate that these regions likely interact with the stem of U6 snRNA substrate to enhance methylation. The VCRs (in the full-length METTL16) significantly increase binding of U6 snRNA to the enzyme and strengthen methylation of U6 snRNA by over two to three orders of magnitude compared to the MTD alone [36]. The arginine-rich region (residues 382–388, Figure 1A,C) is a key element of the VCRs, enhancing methylation and raising the affinity of METTL16 for U6 snRNA. The methylation efficiencies of full-length METTL16 and MTD towards hp1 of MAT2A are almost the same under standard conditions (1 μM substrate concentration) [31,36]. However, the comprehensive steady-state kinetic analyses of the MAT2A hp1 methylation showed that the K_m_ of 0.76 ± 0.1 µM, estimated for MAT2A hp1 with MTD, drops to 0.027 ± 0.05 µM for the full-length METTL16. The VCRs increase affinity between METTL16 and MAT2A hp1 and enhance the methylation of MAT2A hp1 to a lesser extent than in case of U6 snRNA [36]. Different impacts of VCRs on methylation of both RNA substrates could arise from variations in the neighborhood of the RNA methylation sites. More precisely, the U6 substrate is composed of telestem-bulge-internal stem-loop (ISL) structure (Figure 2C). Studies suggest that VCRs interact with the stem of the ISL motif and bend the telestem-bulge-ISL structure [36]. This rearrangement either relaxes the telestem or destabilizes the bulge junction and, in consequence, enables the formation of a quasi-loop, similar to the loop of hp1. Possibly, the interaction between VCRs and the stem of hp1 stabilizes the transition structure of the substrate, which is required for effective catalysis [36].

### 5.2. The RNA Binding Site Is Composed of Positively Charged Residues Important for Methylation

Residues K5, R10, R12, K14, K16, R41, K47, R74 (from N-terminus), as well as R82, R279, and R282 (within the Rossmann fold) contribute to the positively charged grove on the surface of METTL16 [29,32]. Some of those residues are directly involved in RNA binding. K47 and R279 form a claw-like structure [29,32], accommodating the recognition loop of hp1x and hp6 RNA substrates [31]. Substitutions K47E and R279E reduce and abolish methylation, respectively [32]. K47 hydrogen bonds to G within the loop linker of hp1x and hp6. Interactions of R279 are even more conspicuous; the side chain of R279 makes contact with C5 and G7 of hp1x (numbering in Figure 2B), supporting the proper conformation of the CAG motif within the recognition loop of the RNA substrate. These interactions secure the orientation of acceptor adenosine for methyl transfer. Simultaneously, R279 stabilizes the R-loop of METTL16 by interacting with Ser208. However, in the METTL16-MTD/hp6 complex, all of these interactions are absent due to the different orientation of the R279 side chain.

Combined mutations K5A, R10A, R12A, K14A, K16A highly reduced RNA-binding and impaired methylation activity of METTL16, which indicates a role of N-terminus in METTL16/RNA interactions [32]. However, none of these residues make direct contact with RNA in the METTL16-MTD/substrate complexes. Mutations R74E, R82E, and R282E obliterated methylation by METTL16 (Table 1) [31,32]. R282 interacts with the acceptor adenosine (hp1x and hp6), supporting its positioning for methyl transfer. R74 lies above the edge surrounding the coenzyme-binding pocket, while R82 interacts with the methionine moiety of SAM [29,32].

## 6. Comparison of Human m^6^A Methyltransferases Targeting mRNA and ncRNA

The majority of m^6^A marks in human transcriptome are installed by the writer RNA methyltransferase complex composed of the METTL3 catalytic subunit, METTL14, and accessory proteins: WTAP, VIRMA, RBM15, HAKAI, and ZC3H13 [11,12,13,14,15]. METTL3 and METTL14 together form a core of the enzyme. The combination of RNA-binding elements from both subunits contributes to the efficient catalysis of METTL3/METTL14 [69]. The activity of METTL3-MTD also requires the following: (i) two CCCH zinc fingers before the METTL3-MTD [70,71,72], (ii) the RGG domain at the METTL14 C-terminus [73], and (iii) the α-helical motif preceding the catalytically inactive MTD of METTL14 [69]. In contrast, the MTD of METTL16 alone is sufficient for methylation of MAT2A mRNA [16,31]. Nonetheless, the VCRs of the METTL16 C-terminal domain are essential for METTL16/U6 snRNA interaction and for efficient substrate turnover. We also cannot rule out other cellular factors that may affect METTL16 efficiency or specificity.

The METTL3/METTL14 complex co-transcriptionally methylates adenosine within the conserved RRACH motif (R = A or G; H = A, C or U)—preferentially GGACU [10,69,74,75]. Structural requirements for METTL3/METTL14 substrates are still not completely clear. Liu et al. indicated that METTL3/METTL14 methyltransferase recognizes sequence rather than the structure of RNA substrates [10]. However, the most recent study suggests some secondary structure dependence of METTL3/METTL14 targets [69]. The stability of the structure exposing the target sequence seems to be an important determinant of methylation yield. Furthermore, the N-terminal domain (NTD) of METTL3 modulates structure dependence in RNA methylation. It is suggested that the NTD either influences the ability to locally destabilize the structure near the m^6^A modified adenosine or impacts the substrate-binding mode. The interactions between NTD and accessory proteins, e.g., WTAP, may further affect substrate specificity [69]. This modular architecture of METTL3/METTL14 probably allows it to target a broader spectrum of RNAs than METTL16.

Both METTL16 and METTL3/METTL14 methylate mRNAs and ncRNAs. Most of the m^6^A sites installed by METTL3/METTL14 are localized in 3′UTRs, near stop codons [76,77]. VIRMA, one of the METTL3 accessory proteins, mediates this target preference [15]. Ke et al. confirmed the highest concentration of METTL3-related m^6^A sites in 3′UTRs. The authors also suggest localization of numerous m^6^As at the start of the last exons rather than around stop codons. The 3′ terminal exons, including the 3′UTRs, contain 70% of all m^6^A sites in mRNA [78]. Most (82%) of the METTL16-associated m^6^A marks (direct or indirect) are placed in introns or within intron-exon boundaries [16], while 87% of the introns crosslinking to METTL16 are constitutively spliced [17]. The subset of introns containing METTL16-related m^6^A modifications is characterized by higher GC content and shorter length [16]. Interestingly, short GC-rich introns are likely spliced by intron definition; that is, the splicing factors recognize an intronic unit and arrange the splicing machinery across introns [79]. Moreover, splicing-disrupting mutations, associated with short introns spliced by intron definition, may cause intron retention [79,80,81]. As shown for MAT2A pre-mRNA, installation of m^6^A via METTL16 also mediates intron retention near the 3′-end of the transcript, leading to degradation of unspliced mRNA. Notably, the subset of transcripts carrying m^6^A METTL16-dependent sites likely represents intron-retained RNAs [16]. Therefore, it would be interesting to know how other METTL16-associated methylations affect the fate of mRNAs. So far, the mechanistic link between METTL16-associated m^6^A marks and above splicing characteristics is not known.

**Figure 3:**
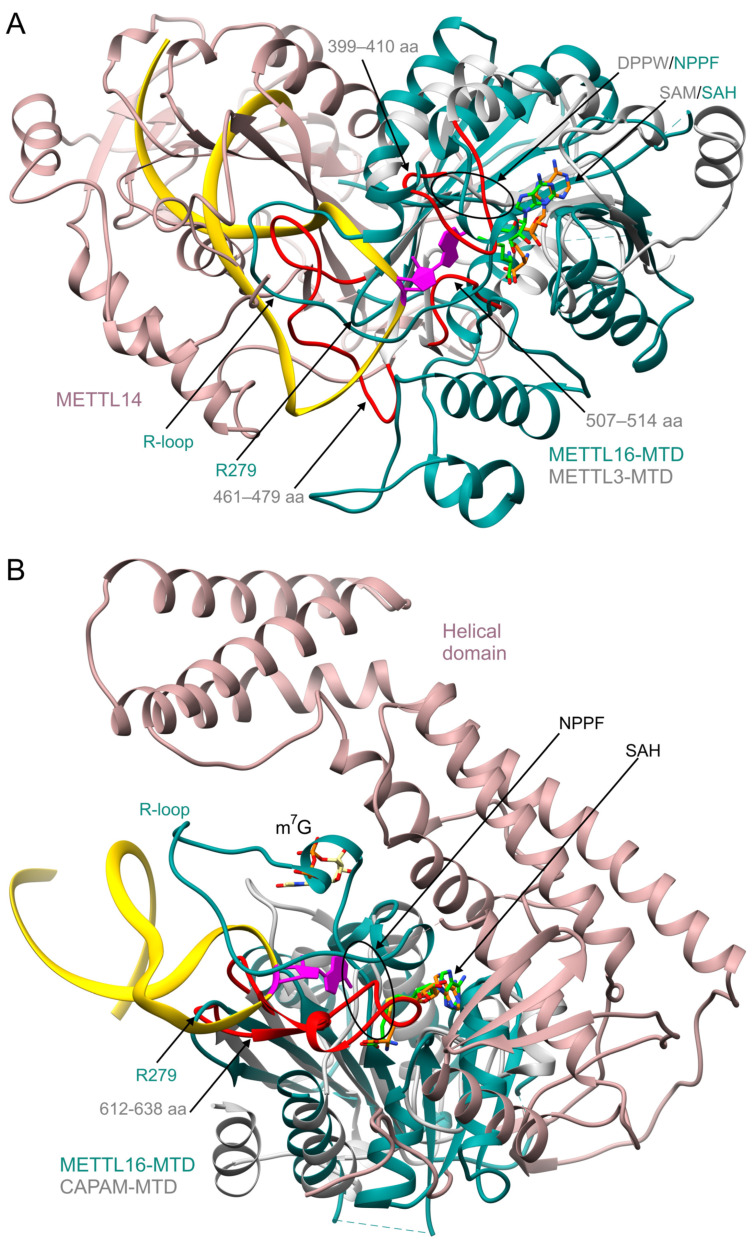
Structural comparison between the MTD domain of METTL16 and the MTDs of human mRNA and ncRNA m^6^A MTases. METTL16-MTD (2–310 aa, turquoise)/hp1x RNA (gold) model (PDB ID: 6du4) was used for superposition with two other human MTases; SAH (orange) was modeled into the coenzyme binding pocket of METTL16-MTD/hp1x using METTL16-MTD/SAH structure (6gfn); the acceptor adenosine is in magenta. Catalytic domains of superimposed MTases are gray; their additional subunits or domains are rosy brown; SAH or SAM are neon green; red indicates structural elements likely contributing to the substrate specificity. Panels present superposition of human METTL16-MTD/hp1x/SAH structure with (**A**) METTL3/METTL14/SAM complex (5il1); (**B**) CAPAM/SAH complex (6irw), residues 2–78 of METTL16 are hidden for clarity, the m^7^G cap is modeled using CAPAM/SAH/m^7^G structure from zebrafish (6irz).

Structural comparison between METTL16 and METTL3/METTL14 reveals similar global architecture of METTL16 and METTL3 catalytic domains, while their sequences share no significant similarity [29,31,70,82]. Superposition of METTL16-MTD in complex with RNA substrate (PDB ID: 6du4) and METTL3-MTD (5il1) has shown that the catalytic motifs, ^184^NPPF^187^ of METTL16 and ^395^DPPW^398^ of METTL3, as well as the coenzyme binding sites, overlap well (Figure 3A). However, the stem structure of the METTL16 substrate (hp1x) would clash with the METTL14 subunit (Figure 3A). It is not surprising, as RNA targets of both enzymes are distinctly different [10,16,31,69]. Three fragments of METTL3, involving residues 399–410, 461–479, and 507–514, map to the same face of the protein structure as the METTL16 RNA-binding site (Figure 3A) [29,31]. Interestingly, the 399–410 and 461–479 fragments of METTL3 correspond to the R-loop and R279 of METTL16, respectively. Given that these elements of METTL16 are essential for interaction with RNA and methylation, equivalent motifs exposed on the METTL3/METTL14 interface may have similar roles but use a different mechanism of substrate recognition.

Cap-specific adenosine methyltransferase (CAPAM) PCIF1 is a newly discovered human m^6^A writer [19,20]. It is recruited to the early elongation complex of RNAPII by the WW domain. The CAPAM specifically recognizes 5′ capped end of RNA transcripts, manifesting m^7^GpppAm motif, and methylates Am co-transcriptionally. The authors speculate that Am fits better than A to the CAPAM catalytic pocket due to the presence of 2′-O-methyl group or/and C3′*endo* ribose [19]. Some (but not strong) sequence specificity of CAPAM has been reported for the 5′-terminal sequence of mRNAs. A 6-mer is the minimal substrate of this enzyme. The role of the m^6^Am mark remains debatable. Akichika et al. have shown that this modification upregulates cap-dependent translation [19]. In contrast, Sendinc et al. proposed that m^6^Am negatively impacts translation, while Boulias et al. suggested a minor effect of the modification on translation but rather on mRNA stability [83,84]. The MTD of human CAPAM (PDB ID: 6irw) has the canonical Rossmann fold, exhibiting a four-stranded β-sheet placed between two clusters of helices. The CAPAM-MTD structure overlaps well with the C-terminal part of METTL16-MTD/RNA Rossmann fold (6du4), including the strands β4-β7 (Figure 1A,B and Figure 3B). The coenzyme binds to the N-terminal portion of the CAPAM Rossmann fold and superimposes well with the coenzyme binding site in the METTL16-MTD/SAH complex (6gfn, Figure 3B). CAPAM has the NPPF putative catalytic motif (residues 553–556), overlapping with the corresponding fragment in METTL16. The small domain of CAPAM, including residues 612–638, clashes with the superposed hp1x of the METTL16-MTD/RNA complex, indicating differences in substrate specificity. In CAPAM, the site corresponding to the METTL16-MTD/RNA R-loop is occupied by the m^7^G cap (superposed from the CAPAM/SAH/m^7^G complex of zebrafish, 6irz, Figure 3B). Residues within the CAPAM-MTD, as well as the helical domain of CAPAM, specifically recognize the m^7^G cap. Moreover, the CAPAM helical domain contains a conserved positively charged groove, which likely functions as the RNA-binding surface [19]. Overall, structural elements differentiating m^6^A MTases, such as the helical domain of CAPAM, the R-loop, and the N-terminus of METTL16, define substrate specificity.

## 7. Comparison of METTL16 and rRNA Methyltransferases

RlmF, installing a m^6^A mark in 23S rRNA (A1618) of *E. coli* [24], shares 31% identity and 50% similarity with the MTD of METTL16 [29]. RlmF confers m^6^A mark in rRNA ACAGR motif, matching the pattern of U6 snRNA and MAT2A substrates of METTL16 [16,24].

**Figure 4:**
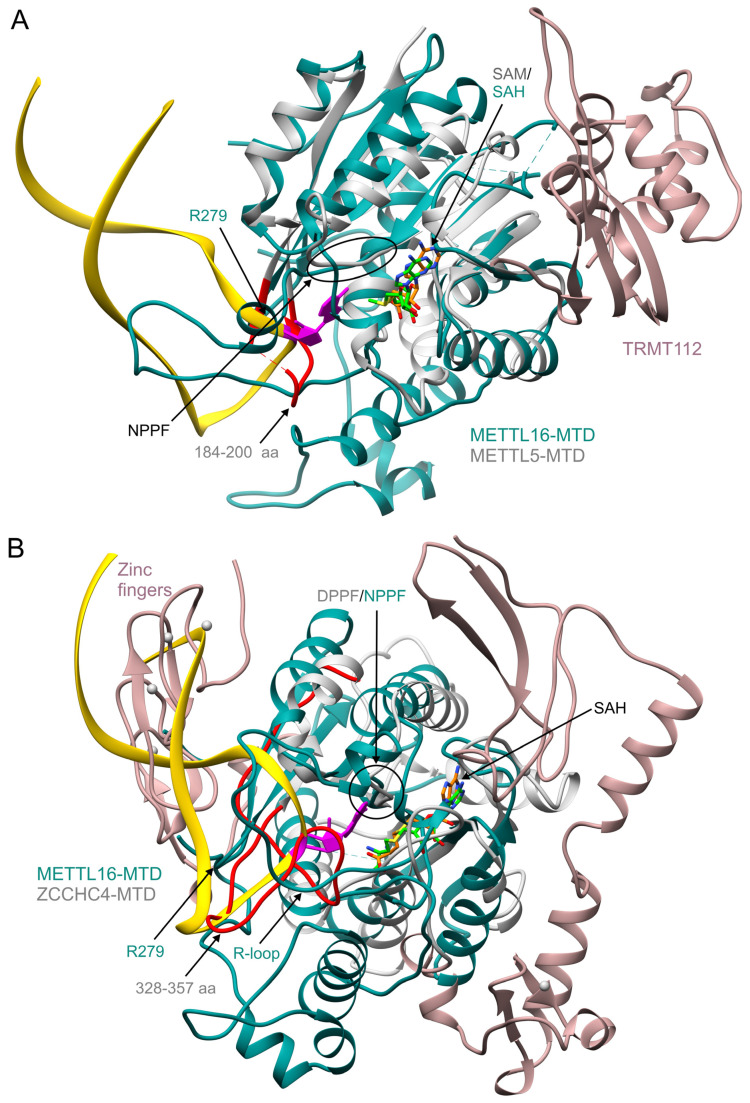
Structural comparison between the MTD domain of METTL16 and the MTDs of human rRNA m^6^A MTases. All structural elements are color-coded as in Figure 3. Panels present superposition of human METTL16-MTD/hp1x/SAH structure with (**A**) METTL5/TRMT112/SAM complex (6h2u); (**B**) ZCCHC4 /SAH structure (6uca).

Recently, two human rRNA methyltransferases METTL5/TRMT112 and ZCCHC4, have been reported to confer m^6^A marks within sequence UAACR at positions 1832 in 18S and 4220 (alternatively referred to as A4190) in 28 rRNA, respectively. Both methyltransferases localize to nucleoli, the site of rRNA biogenesis [21,22,23,85,86]. METTL5/TRMT112 is the second human m^6^A methyltransferase, after METTL3/METTL14, working as a heterooligomer. METTL5 is the catalytic subunit of the complex, whereas TRMT112 probably contributes to RNA binding and likely activates METTL5, stimulating its interaction with SAM. Both proteins contact each other through a parallel β-zipper. TRMT112 masks a large hydrophobic patch on the surface of METTL5 and stabilizes it [21]. METTL5 has a Rossmann fold of class I SAM-dependent MTases and, similarly to METTL16 and CAPAM, contains the conserved NPPF putative catalytic motif (residues 126–129). The MTDs of METTL5 (PDB ID: 6h2u) and METTL16 (6du4) share the same topology (Figure 4A). The putative catalytic residues of METTL5 and METTL16 superpose perfectly. However, other residues coordinating the acceptor adenosine in METTL16 are not conserved in METTL5. The residues 184–200 of METTL5 (corresponding to a short loop of METTL16, exposing R279) would clash with hp1x RNA (Figure 4A), confirming that substrate specificity differs for both enzymes. Moreover, based on similarities with DNA MTases, METTL5/TRMT112 probably extrudes the acceptor adenosine from dsRNA; thus, its RNA-binding mode differs noticeably from that of other m^6^A RNA MTases [21].

ZCCHC4 is the second human rRNA m^6^A methyltransferase known to date [21,22]. A stem-loop structure, observed in the 28S rRNA subunit, is the substrate of this methyltransferase; however, sequential or structural preferences of ZCCHC4 are not clear [22,85]. The catalytic domain of ZCCHC4, in the central part of the enzyme sequence, reveals the Rossmann fold (Figure 4B). Superposition of the MTDs of ZCCHC4 (6uca) and METTL16 shows a similar global architecture and good superimposition of putative catalytic motifs. This means that the good overlap of putative catalytic residues and the coenzyme position is a universal feature for all presented MTases, METTL16, METTL3, CAPAM, METTL5, and ZCCHC4 (Figure 3 and Figure 4). ZCCHC4 contains the DPPF putative catalytic fragment (residues 276–279). Compared to the MTD of METTL16, ZCCHC4 lacks the β6-strand within the Rossmann fold (using default UCSF Chimera parameters; Figure 1A,B and Figure 4B). This element’s place is occupied by a long loop (mapping residues 328–357), which sterically clashes with the superposed hp1x of the METTL16-MTD/RNA model. The C-terminus of ZCCHC4, rich in Cys and His residues, forms zinc fingers mapping to the same surface area of ZCCHC4 as the hp1x stem in its complex with METTL16-MTD (Figure 4B). The superposition indicates that both the fragment 328–357 and zinc fingers of ZCCHC4 may be essential for dictating RNA specificity. 

## 8. The Proposed Mechanism of Methyl Transfer Catalyzed by METTL16

**Figure 5:**
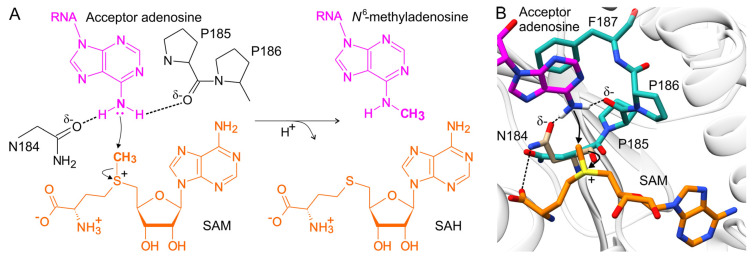
The mechanism of methyl transfer catalyzed by METTL16. (**A**) A graphical representation of methyl transfer mechanism from SAM to the N6 atom of acceptor adenosine ([29], figure modified). (**B**) To propose the course of the methyl transfer reaction, SAM (orange stick representation) was modeled into the SAH binding site of METTL16-MTD/SAH (2h00) and superimposed with the METTL16-MTD/hp1x complex (6du4; white hydrogen atoms are shown only for the N6 amino group). The catalytic motif of METTL16-MTD/hp1x is turquoise, F187 is in an optimal position to stack with the acceptor adenosine. The proper position of the N184 side chain (tan) is coordinated by SAH/SAM carboxyl and present only in the METTL16/SAH complex.

m^6^A methyltransferases contain the conserved motif [DNSH]PP[YFW] located near the SAM binding pocket and the methylated adenosine [39,87,88]. Residues within the NPPF region of METTL16 are essential for m^6^A methylation, as METTL16 N184A, PP185/186AA, and F187G mutants were not able to methylate U6 and MAT2A RNA substrates in vitro (Table 1) [16,31]. Here, I present a model of methyl transfer reaction based on the published structure of METTL16-MTD/hp1x complex (PBD ID:6du4) and METTL16-MTD/SAH (2h00); SAM position was inferred from SAH. First, the acceptor adenosine is positioned in the active site of METTL16. The proper placement of adenosine is conditioned by π-π stacking with the aromatic side chain of F187. The N6 amino group of the adenosine is negatively polarized by the N184 Oδ atom and the P185 carbonyl oxygen of METTL16 through hydrogen bonding. As an effect of the N6 amino group polarization, it withdraws the methyl group from SAM via an S_N_2 mechanism (Figure 5A) [29,88]. Notably, the Oδ atom of N184 and N6 amino group of acceptor adenosine in METTL16-MTD/RNA complex locate ~5 Å from one another—too far for hydrogen bonding. However, in the METTL16-MTD/SAH complex (PDB ID: 2h00), the N184 side-chain conformation is different, placing the N184 Oδ atom and N6 amino group of adenosine within a 3-Å distance—short enough for an interaction. The conformation of the N184 side chain favoring hydrogen bonding with adenosine is forced by the interaction between the amino group of N184 and the carboxylic group of SAH/SAM (Figure 5B). The same orientation of the N184 side chain was observed in other crystal structures of METTL16-MTD in complex with SAH (PDB ID: 6gfk, 6gfn) [32].

The conformation of the F187 side chain also varies between METTL16-MTD/SAH and METTL16-MTD/RNA [31]. In METTL16-MTD/RNA complex, F187 stacks with the acceptor adenosine and secures it for methyl transfer (Figure 5B). F187 in the METTL16-MTD/SAH complex (followed by a disordered R-loop) is shifted and oriented differently. It is possible that F187 π-π stacks with the acceptor adenosine before its binding and that both enter the catalytic pocket together. In conclusion, the first and last residues of the NPPF catalytic motif, N184 and F187, are dynamic, whereas conformation of the motif that is proper for catalysis requires binding of both the RNA substrate and the coenzyme. This supports the hypothesis that various regions of METTL16 rearrange to ensure the optimal fit between the enzyme, the coenzyme and the RNA substrate.

## 9. METTL16 in Cancer

The disruption of the m^6^A pattern alters multiple biological processes and might result in cancer development and its progression when it affects tumor-related genes. Consistently, m^6^A has been associated with tumor proliferation, differentiation, tumorigenesis, invasion, metastasis, and chemoradiotherapy resistance. Moreover, m^6^A may function as an oncogene or anti-oncogene in malignant tumors. The m^6^A epitranscriptome regulators have been linked with multiple types of cancers [89,90,91,92]. 

METTL16 is an essential protein for mammals. Attempts of the *METTL16* knockout in cell lines and mice failed or resulted in embryonic lethality, respectively [16,32]. Several reports indicate an association of METTL16 with some types of cancers. Mutations of crucial METTL16 residues, such as R200Q or G110C (Table 1), have been reported in large intestinal cancer, potentially implicating METTL16 in the disease [31,93,94]. The expression level of METTL16, together with other m^6^A regulators, such as METTL3, METTL14, FTO, and ALKBH5, affect the outcome of colorectal cancer (CRC) patients [95]. In CRC with high microsatellite instability, frameshift mutations of genes involved in methylation, including *METTL16*, may lead to tumorigenesis via their inactivation [96]. Copy number variations of several m^6^A regulatory genes, including *METTL16*, influence overall survival rate of patients with soft-tissue sarcoma [97]. Recently, downregulation of METTL16 has been correlated with poor overall survival in patients with hepatocellular carcinoma (HCC) and endocrine system tumors [98,99]. Decreased expression of METTL16 is linked with activation of numerous metabolic pathways in HCC, suggesting a possible role of METTL16 in metabolic reprogramming–a hallmark of cancer [99]. The METTL16 low expression has also been determined in ovarian cancer (OC) [100]. Thus, *METTL16* is considered a protective gene, suppressing the development of OC, HCC, and endocrine system tumors [98,99,100]. However, the high expression of METTL16 has been linked with poor survival rate in patients with breast cancer [101]. Finally, METTL16 is known to specifically recognize oncogenic lncRNA, MALAT1. The role of this interaction remains unknown; however, there are speculations suggesting that METTL16/MALAT1 interaction may be related to oncogenesis [42].

## 10. METTL16 Remains Enigmatic

Our knowledge about METTL16 has expanded significantly during the last five years. We have learned that this protein interacts with multiple RNAs: pre-mRNAs, ncRNAs, lncRNAs, rRNAs. METTL16, next to the four other enzymes: METTL3/METTL14, CAPAM, METTL5/TRMT12, and ZCCHC4, functions as a human SAM-dependent m^6^A methyltransferase. It installs m^6^A marks on at least two substrates, U6 snRNA and 3′ end hairpins of MAT2A pre-mRNA. METTL16 manifests a rigorous sequential and structural basis of substrate recognition. New structural information regarding this enzyme has been recently revealed, for instance, discovering its autoregulatory function. Last year, we got our first insight into the structure of the METTL16 C-terminal domain. Despite all these fascinating discoveries, METTL16 still keeps secrets, and numerous questions about this protein need to be addressed. For instance, how to explain the broad METTL16 impact on m^6^A pattern of transcriptome, not entirely mediated by the SAM level regulation? Are there other RNA substrates of METTL16? May unidentified cellular factors expand substrate specificity of METTL16? Does METTL16 universally function as both modification writer and reader, e.g., in splicing regulation? Are there other, methylation-independent, biological functions involving METTL16/RNAs interactions? These and many other questions concerning METTL16 are awaiting answers, opening a broad field for further studies.

## Figures and Tables

**Table 1:** Significance of METTL16 residues. Blue indicates residues within the N-terminus (1–78), gray indicates residues within the Rossmann fold (79–288), yellow indicates residues from the C-terminal domain.

METTL16 Residue(s)	Significance	Mutation(s)	Effect of Mutation(s)	Reference
1–39	probable interaction with RNA	Deletion	abolishes RNA binding	[32]
K5	probable interaction with RNA	K/E	reduces methylation	[32]
R10	probable interaction with RNA	R/E, R/D	reduces methylation	[32]
K5, R10, R12, K14, K16	probable interaction with RNA	Single mutations to A	not effect on methylation	[32]
K5, R10, R12, K14, K16	probable interaction with RNA	Combined mutations to A	highly reduce RNA binding, abolish methylation	[32]
K26, K31	-	Combined mutations to A	neutral, no effect on methylation	[32]
N39	interaction with RNA	N/A	neutral, no significant effect on methylation	[31]
K47	interaction with RNA, formation of claw-like structure	K/E	reduces methylation	[32]
R74	an element of RNA-binding groove	R/E	abolishes methylation	[32]
R82	interaction with SAH/SAM (Figure 1D)	R/A	abrogates methylation	[31,32]
G110	an element of conserved GXG motif, interaction with SAH/SAM (Figure 1D)	G/C	abrogates methylation	[31]
E133	a conserved element of SAM-MTases, interaction with SAH/SAM (Figure 1D)	E/A	abrogates methylation	[31]
K163, M167	elements of K-loop, autoinhibitory activity of METTL16 (Figure 1E)	K/A, M/A	little effect on RNA affinity, increase methylation, K163A reduces MAT2A splicing and level of MAT2A mRNA	[31]
N184	an element of catalytic NPPF motif, interaction with acceptor adenosine and SAH/SAM (Figure 5)	N/A	abrogates methylation, increases MAT2A splicing and level of MAT2A mRNA	[31]
P185-P186	an element of the catalytic NPPF motif, interaction with acceptor adenosine (Figure 5)	PP/AA	reduces RNA affinity, abolishes methylation	[16,32]
F187	an element of catalytic NPPF motif, stacking with acceptor adenosine (Figure 5)	F/G	abolishes RNA binding and methylation	[16,32]
190–218	modulation of methylation activity	Deletion	no significant effect on RNA binding, abolishes methylation	[32]
R200, R203, R204	modulation of methylation activity	Combined mutations to E	reduces RNA binding, abolishes methylation	[32]
R200	interaction with the transition region of hp1 (G1, G9—Figure 1F and Figure 2B), modulation of methylation activity	R/Q	no significant effect on RNA affinity, increases methylation, reduces MAT2A splicing and level of MAT2A mRNA	[31]
R279	interaction with RNA, formation of claw-like structure	R/E	abolishes methylation	[32]
K310/R541, W378/H380, R552/R557	Conserved residues in vertebrate-conserved regions (VCRs)	K/R to A/A, W/H to A/A, R/R to A/A	neutral, no significant effect on U6 snRNA methylation	[36]
Arginine-rich region, 382–388	Conserved residues in VCRs, interactions with U6 snRNA	Deletion	reduces RNA affinity, decreases of U6 snRNA methylation	[36]

## Data Availability

Not applicable.
